# A Novel *C*3/*C*4-Fused Indole Scaffold through Acid-Catalyzed Cascade Reaction

**DOI:** 10.3390/molecules29133064

**Published:** 2024-06-27

**Authors:** Felix Potlitz, Gottfried J. Palm, Anja Bodtke, Michael Lammers, Dennis Schade, Andreas Link

**Affiliations:** 1 Institute of Pharmacy, University of Greifswald, 17489 Greifswald, Germany; felix.potlitz@gmail.com (F.P.); bodtke@uni-greifswald.de (A.B.); 2 Institute of Biochemistry, University of Greifswald, 17489 Greifswald, Germanymichael.lammers@uni-greifswald.de (M.L.); 3 Institute of Pharmacy, Department of Pharmaceutical and Medicinal Chemistry, Christian-Albrechts-University of Kiel, 24118 Kiel, Germany; schade@pharmazie.uni-kiel.de; 4 Partner Site Kiel, DZHK, German Center for Cardiovascular Research, 24105 Kiel, Germany

**Keywords:** indoles, cascade reaction, chemical diversity

## Abstract

3,4-bridged indoles are underrepresented among the vast number of indoles described in the literature. Attempts to access 3,4-macrocyclized indoles led to the unexpected formation of a novel tetracyclic indole through intramolecular acid-catalyzed ring contraction. The herein-established one-step synthetic route provides an excellent medicinal chemistry platform for the construction of screening libraries covering a unique chemical space of indoles.

## 1. Introduction

**Figure 1:**
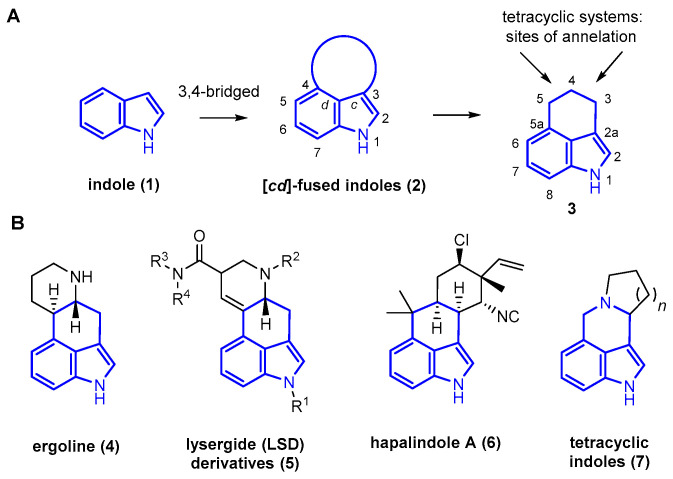
3,4-bridged indoles with 6-membered [*cd*]-annelants giving rise to tetracycles via two distinct sites of annelation (**A**), and examples of corresponding polycyclic indole heterocycles (**B**).

**Figure 2:**
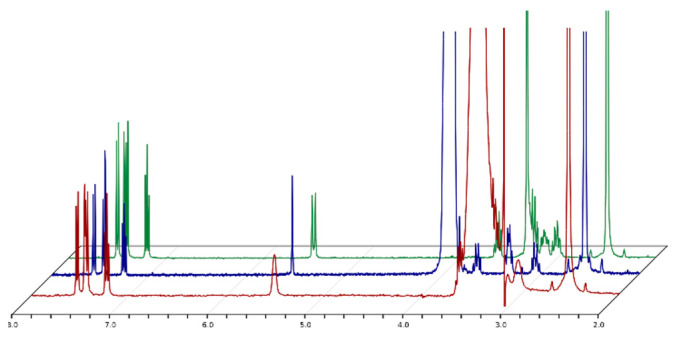
Comparison of ^1^H-NMR spectra of the novel indole **12** under various conditions: (green) in a low concentration; (blue) after proton exchange by D_2_O treatment; and (red) after selective irradiation resulting in decoupling of the NH signal.

Fused derivatives of the naturally occurring heterocycle indole (Figure 1A, **1**) play an important role in medicinal chemistry as a skeleton of highly bioactive natural products, semi-synthetically modified alkaloids, and pharmaceuticals [1,2,3]. [*a*]- or [*b*]-annelated indoles can straightforwardly be obtained, for example, via palladium-catalyzed double-alkylation approaches, *C*3/*C*4-annelated (i.e., [*cd*]-fused) indoles (Figure 1A, **2**) are less easily accessible [4,5,6,7]. This circumstance likely explains why such [*cd*]-fused indoles are, in general, underrepresented within the overall chemical space of indoles.

However, several prominent natural products exist that contain 3,4-bridged indoles with 6-, 7-, 8-, or higher-membered annelants, oftentimes as part of even more complex, polycyclic scaffolds. Among these, tetracyclic heterocycles (Figure 1A, general structure **3**) comprise the well-known ergot alkaloids (e.g., ergoline **4**), including lysergide (LSD) derivatives (Figure 1B, **5**). While LSD itself is pharmacologically highly potent, the receptor subtype promiscuity for various serotonin receptors, and thus, the inherent lack of clinical safety, of this molecule stands heavily in the way of its therapeutic use.

Interestingly, the majority of reported tetracyclic indoles derived from [*cd*]-fused **3** are annelated at the 4,5-bond (Figure 1A). Only very few examples exist for [*cd*]-fused indole tetracycles with annelants at the 3,4-bond, such as the cyanobacteria-derived class of hapalindoles (Figure 1B, **6**) that exhibit diverse pharmacological activities [8].

The synthesis of condensed indole-containing heterocycles is often a challenging task, usually requiring multi-step sequences [8,9]. Multiple approaches to access *C*3/*C*4-annelated indole scaffolds have been reported to date, oftentimes exploiting the inherent reactivity of the indole ring as a platform for chemical diversity [7]. The nucleophilic *C*3-position allows different types of alkylation reactions, for instance, with various Michael acceptors [10]. Such *C*3-functionalizations enable consecutive bond-forming reactions in cascade-, tandem-, or domino-type sequences yielding a small but diverse variety of condensed heterocycles. Other strategies involving the construction of the indole ring later in the course of the reaction, e.g., during cascade reactions, exist as well but were not considered in this work.

Notably, the Seidel group described an elegant approach to [*cd*]-fused tetracyclic indoles with annelants at the 3,4-position (Figure 1B, **7**) from 1*H*-indole-4-aldehyde by annelation reaction via the intermediate formation of azomethine ylides [11]. Still, the described **7**-type fused indoles are scarce, and thus, they are exciting to be explored by means of medicinal chemistry for drug discovery campaigns.

## 2. Results

**Scheme 1:**
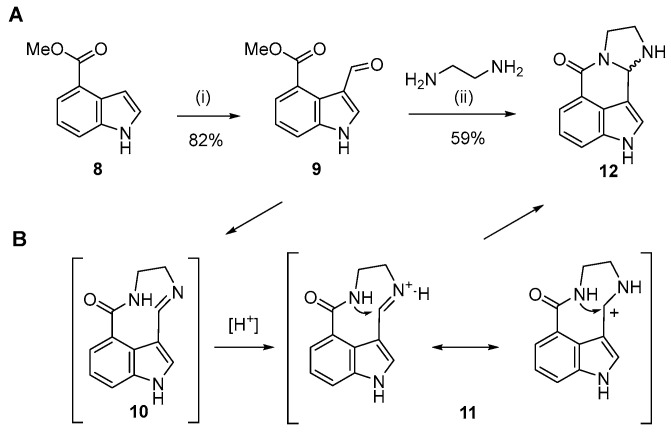
Synthesis of novel tetracyclic indole **12** (**A**) and illustration of a postulated reaction mechanism comprising intermediates **10** and **11** (**B**). (i) Phosphoryl chloride (1.1 equiv.) in DMF, 0–22 °C, 3 h; water; 82% isolated yield. (ii) Ethane-1,2-diamine (3.0 equiv.), acetic acid (1.5 equiv.), methanol, 60 °C, 30 min; 59% isolated yield.

We, herein, report on a serendipitous finding that readily provided access to an unprecedented tetracyclic indole ring system, 8,9,10,10a-tetrahydroimidazo[1,2-*b*]pyrrolo[4,3,2-*de*]isoquinolin-6(2*H*)-one (**12**) (Scheme 1A) [9].

Starting from a commercially available 1*H*-indol-4-carboxylic acid methyl ester (**8**), the intermediate 3-formyl-1*H*-indol-4-carboxylic acid methyl ester (**9**) was obtained in a very good yield by applying a modified Vilsmeier–Haack reaction. The formylated intermediate **9** was then heated with acetic acid and ethane-1,2-diamine in methanol to give crude product **12** in a fair yield. After crystallization from water, an analytically pure sample was obtained.

Given that the formation of product **12** was not planned, the structure of the obtained crystalline compound had to be elucidated. Initially, we envisioned and anticipated the formation and isolation of the hypothetical 3,4-macrocyclized indolophane **10** (Scheme 1). This imine would have been expected to display a proton signal corresponding to the secondary amide. However, no such signal could be identified in the ^1^H-NMR spectrum of putative **10** in the expected range of chemical shift of about 6–10 ppm (Figure 2).

Since the expected molar mass was confirmed via ESI high-resolution accurate mass spectrometry in positive mode, we hypothesized that a constitutional isomer had been formed. Further investigation of the reaction conditions led to the mechanism proposed in Scheme 1B.

Due to the presence of stoichiometric amounts of acetic acid in the reaction mixture, and the assumption that the imine-containing indolophane intermediate **10** formed via ring closure as intended, the protonation of the weakly basic imino group under these conditions appears to be feasible [12]. The resulting carbenium-iminium species **11** in close proximity to the amide moiety is expected to favor a nucleophilic attack by the amide-nitrogen (Scheme 1B). Although the amide group is usually not known to be a good nucleophile, the reaction seems well possible under acid catalysis, resulting in the unexpected formation of tetracyclic indole derivative **12**.

**Figure 3:**
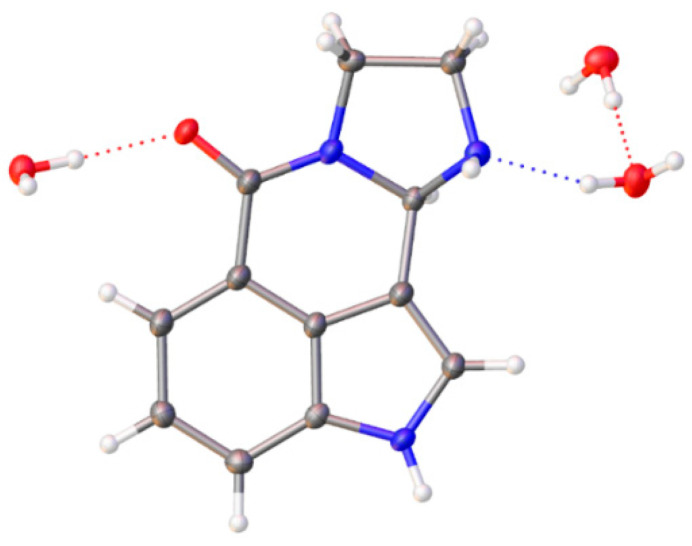
X-ray structure of *R*-enantiomer of product **12** obtained as a crystalline racemate from water.

Albeit both the postulated reaction mechanism and structure of **12** are suitable to explain the absence of an amide proton signal, we sought unambiguous proof by obtaining an X-ray structure. Thus, indole **12** was repeatedly recrystallized from boiling water to acquire crystals suitable for X-ray diffractometry. The results from the X-ray diffraction pattern are in excellent agreement with racemic **12** as proposed, proving the formation of both enantiomers to be present in the crystal. Figure 3 shows the structure of the *R*-enantiomer.

## 3. Discussion

**Figure 4:**
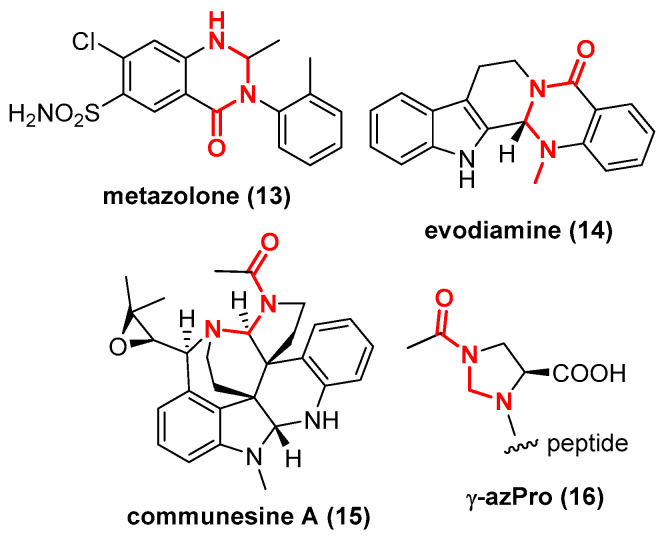
Structures of pharmaceutical agents and natural products with *N*-acyl aminal motifs (red) comparable to the newly introduced [*cd*]-fused tetracyclic indole **12**.

It is textbook knowledge that two C-N single bonds on an sp^3^-hybridized C-atom are energetically unfavorable in comparison with one C=N double bond, and for this reason and electronic repulsions, many aminals tend to be unstable. For this reason, we looked for stable aminals in the literature in order to find arguments in favor of our hypothetical structure **12** before the X-ray data were obtained. We found that the new structure obtained in **12** appeared striking to us at first glance, but this structural motif, indeed, is not unprecedented. As a result of our literature search, we conclude that the cyclic *N*-acyl aminal in **12** appears to be stabilized by the inclusion into a heterocyclic ring system and the absence of a proton on the β-*C* atom, which would otherwise enable enamine formation via β-elimination. Pharmaceutical agents such as the thiazide-like diuretic metazolone (Figure 4, **13**) or natural products with this structural motif (e.g., evodiamine **14** in Figure 4) comprise similar and reasonably stable aminals. Even exocyclic *N*-acyl moieties seem to be tolerated regarding chemical stability, as exemplified by communesines A-G (see **15** in Figure 4) and synthetic pH-responsive γ-azaproline (γ-azPro, **16**, in Figure 4) derivatives. The latter has been studied as proline analogs [13,14]. Notably, the sp^2^-character of the *N*γ atom has been reported for collagen model peptides bearing *N*-acylated γ-azPro residues, a finding that perfectly matches the observed planarity in the X-ray structure of the herein disclosed **12**.

In conclusion, we serendipitously discovered an acid-catalyzed cascade reaction that furnished a hitherto unknown, complex tetracyclic indole skeleton in a one-step and atom economic fashion from commercially available material in a fair yield. The new tetracyclic indole scaffold in **12** could serve as a platform for chemical diversity in unbiased cellular and biochemical screenings as well as virtual screening approaches. The latter would allow generating a vast number of hypothetical analogs and derivatives in silico and, once predicted to be active, could easily be synthesized on demand for biological validation [15].

## 4. Materials and Methods

### 4.1. General Information

All starting materials, reagents, and solvents were commercially available and purchased from VWR (VWR International GmbH, Darmstadt, Germany), Abcr (Abcr GmbH, Karlsruhe, Germany), or Carl Roth (Carl Roth GmbH + Co. KG, Karlsruhe, Germany). Unless otherwise stated, the starting materials were used as provided. Thin-layer chromatography on an analytical scale was performed using silica gel 60 F_254_ aluminum plates supplied by Merck (Merck KgaA, Darmstadt, Germany), and visualization was accomplished using UV light (254 nm). NMR analyses were run using a Bruker (Bruker Biospin GmbH, Rheinstetten, Germany) Avance III instrument at 400 MHz (^1^H) and 101 MHz (^13^C), respectively, using DMSO-*d*_6_ as the solvent. Chemical shifts were given in relation to the internal standard tetramethylsilane and reported as parts per million (ppm). A Bruker (Bruker Daltonics GmbH Co. KG, Bremen, Germany) compact or maXis LC-QTOF-MS, operated with ESI ionization, was used to measure the HRAM-MS data. Melting points were determined using an automated Büchi (Büchi Labortechnik GmbH, Essen, Germany) Melting Point M-565 device.

### 4.2. General Experimental Procedures for Synthesis of Compounds ***9*** and ***12*** (Scheme 1)

Methyl 3-formyl-1*H*-indole-4-carboxylate (**9**): To a round-bottom flask, *N*,*N*-dimethylformamide (20.0 mL) and methyl 1*H*-indole-4-carboxylate (**8**) (3.50 g, 20.00 mmol, 1.00 eq.) were added, and the flask was cooled to 0 °C using an ice bath. With vigorous stirring phosphoryl chloride (2.06 mL, 3.37 g, 22.00 mmol, 1.10 eq.) was slowly added dropwise, maintaining the temperature of the reaction mixture below 4 °C. Following the addition, the cooling was removed, and the reaction mixture was stirred at 20 °C for 12 h. The reaction mixture was poured into a saturated aqueous solution of sodium bicarbonate and stirred for 15 min at 20 °C. The pH was brought to 12 by the addition of a 5 M aqueous solution of sodium hydroxide. The precipitate was collected using vacuum filtration via a fritted funnel, washed multiple times with water, and dried, yielding 3.32 g (82%) of a slightly brown amorphous solid. Mp.: 134.2 °C. *R*_f_ = 0.50 (ethyl acetate). ^1^H-NMR (400 MHz, DMSO-*d*_6_): δ (ppm) = 12.49 (s, 1H), 10.21 (s, 1H), 8.36 (s, 1H), 7.76 (dd, *J* = 8.0, 1.0 Hz, 1H), 7.60 (dd, *J* = 7.4, 1.0 Hz, 1H), 7.35 (t, *J* = 7.6 Hz, 1H), and 3.88 (s, 3H). ^13^C-NMR (101 MHz, DMSO-*d*_6_): δ (ppm) = 186.2, 168.7, 137.9, 136.8, 124.7, 123.2, 122.4, 121.3, 118.2, 116.4, and 51.9.
8,9,10,10a-Tetrahydroimidazo[1,2-*b*]pyrrolo[4,3,2-*de*]isoquinolin-6(2*H*)-one (**12**): To a round-bottom flask, methanol (8.0 mL), methyl 3-formyl-1*H*-indole-4-carboxylate **9** (203 mg, 1.00 mmol, 1.00 eq.), acetic acid (86 μL, 90 mg, 1.50 mmol, 1.50 eq.), and 1,2-ethylenediamine (200 μL, 180 mg, 3.00 mmol, 3.00 eq.) were added and stirred at 60 °C for 30 min. The reaction mixture was cooled in an ice bath, and the resulting precipitate was collected via vacuum filtration. It was recrystallized from boiling water, cooled, filtered off, and dried, yielding 126 mg (59%) of the title compound as yellow platelets. Mp.: 264.1 °C. *R*_f_ = 0.27 (5% methanol and 1% triethylamine in dichloromethane). ^1^H-NMR (400 MHz, DMSO-*d*_6_): δ (ppm) =11.35 (s, 1H), 7.51 (dd, *J* = 8.1, 0.4 Hz, 1H), 7.46–7.39 (m, 2H), 7.21 (dd, *J* = 7.9, 7.4 Hz, 1H), 5.50 (s, 1H), and 3.38–3.14 (m, 5H). ^13^C-NMR (101 MHz, DMSO-*d*_6_): δ (ppm) = 160.8, 133.1, 127.2, 122.4, 121.9, 121.3, 115.8, 114.5, 106.9, 73.8, 44.0, and 42.9. HRAMS (ESI, *m*/*z*): calc. for [C_12_H_11_N_3_O+H]^+^: 214.0975; found: 214.0974.
Single-crystal structure determination of (**12**): A crystal was mounted with a 0.1 mm LithoLoop (Molecular Dimensions, Rotherham, UK) under a thin film of paraffin oil. Data were collected from a thin colorless plate on a single-crystal XtaLAB Synergy diffractometer acquired from Rigaku (Neu-Isenburg, Germany) with a Photon-Jet S Cu radiation (λ = 1.54184 Å) source, a four-circle goniometer, and a Hybrid Photon Counting Detector (HyPix6000). Data reduction, including absorption correction, was performed using CrysAlisPro 1.171.42.61a (Rigaku OD, 2022). The structure was solved by direct methods with SHELXS and refined by full-matrix least-squares techniques using SHELXL [16,17]. All non-hydrogen atoms were refined with anisotropic displacement parameters. Hydrogen atoms were refined isotropically at calculated positions using a riding model with their U_iso_ values constrained to 1.2 times the U_eq_ of their pivot atoms for aromatic or 1.5 times the U_eq_ for all other carbon atoms. The amide nitrogen is sp^2^-hybridized. Only the annelated carbon of the tetrahydroimidazole is chiral. Comparable chemistry in alkylated γ-azaprolines, as recently described by Aronoff et al., stabilizes the *N*-acyl aminal [13]. Due to space group C2/c, the crystals contain the racemate of the compound. Each asymmetric unit, though, includes both enantiomers as alternative conformations of the tetrahydroimidazole ring in a ratio of ca. 0.7:0.3. Crystallographic data were deposited into the Cambridge Crystallographic Data Centre, CCDC, 12 Union Road, Cambridge CB21EZ, UK. These data can be obtained free of charge by quoting the depository number CCDC 2284158 by FAX (+44-1223-336-033), email (deposit@ccdc.cam.ac.uk), or their web interface (at http://www.ccdc.cam.ac.uk).

## Data Availability

The original contributions presented in this study are included in this article/the Supplementary Materials. Further inquiries can be directed to the corresponding author.

## Supplementary Materials

The following supporting information can be downloaded at https://www.mdpi.com/article/10.3390/molecules29133064/s1. Table S1: Crystallographic data for compound **12** (PDF). Figure S1: Copies of ^1^H and ^13^C NMR spectra for compound **9**. Figure S2: Copies of ^1^H and ^13^C NMR spectra for compound **12**. (PDF).

## References

[1] Mathada B.S., Yernale N.G., Basha J.N. (2023). The Multi-Pharmacological Targeted Role of Indole and its Derivatives: A review. ChemistrySelect.

[2] Sravanthi T.V., Manju S.L. (2016). Indoles*—*A promising scaffold for drug development. Eur. J. Pharm. Sci..

[3] Kumari A., Singh R.K. (2019). Medicinal chemistry of indole derivatives: Current to future therapeutic prospectives. Bioorg. Chem..

[4] Gao Y.D., Li J.H., Bai S.L., Tu D.Q., Yang C., Ye Z.W., Hu B.C., Qi X.B., Jiang C. (2020). Direct synthesis of annulated indoles through palladium-catalyzed double alkylations. Org. Chem. Front..

[5] Das K.K., Panda S. (2023). 1,2-Metallate Rearrangement Using Indole Boronate Species to Access 2,3-Diarylindoles and Indolines. Org. Lett..

[6] Singh A., Heer S., Kaur K., Gulati H.K., Kumar N., Sharma A., Singh J.V., Bhagat K., Kaur G., Kaur K. (2022). Design, synthesis, and biological evaluation of isatin-indole-3-carboxaldehyde hybrids as a new class of xanthine oxidase inhibitors. Arch. Pharm..

[7] Connon R., Guiry P.J. (2020). Recent advances in the development of one-pot/multistep syntheses of 3,4-annulated indoles. Tetrahedron Lett..

[8] Hohlmann R.M., Sherman D.H. (2021). Recent advances in hapalindole-type cyanobacterial alkaloids: Biosynthesis, synthesis, and biological activity. Nat. Prod. Rep..

[9] Pfaffenbach M., Roller A., Gaich T. (2016). Synthesis of Indolophanes by Photochemical Macrocyclization. Chem.-Eur. J..

[10] Wei D.S., Ni Y., Ma D.W. (2018). Thiourea-Catalyzed Asymmetric Michael Addition of Carbazolones to 2-Chloroacrylonitrile: Total Synthesis of 5,22-Dioxokopsane, Kopsinidine C, and Demethoxycarbonylkopsin. Angew. Chem. Int. Ed..

[11] Zhang C., Das D., Seidel D. (2011). Azomethine ylide annulations: Facile access to polycyclic ring systems. Chem. Sci..

[12] Webber S.E., Canan-Koch S.S., Tikhe J., Thoresen L.H. (2002). Tricyclic Inhibitors of Poly(ADP-ribose) Polymerases. U.S.Patent.

[13] Aronoff M.R., Egli J., Menichelli M., Wennemers H. (2019). γ-Azaproline confers pH responsiveness and functionalizability on collagen triple helices. Angew. Chem. Int. Ed..

[14] Dong J., Xia Q., Yan C., Song H., Liu Y., Wang Q. (2018). C(sp^3^)−H Azidation Reaction: A Protocol for Preparation of Aminals. J. Org. Chem..

[15] Potlitz F., Link A., Schulig L. (2023). Advances in the discovery of new chemotypes through ultra-large library docking. Exp. Opin. Drug Discov..

[16] Sheldrick G.M. (2015). SHELXT—Integrated space-group and crystal-structure determination. Acta Cryst. A.

[17] Sheldrick G.M. (2015). Crystal structure refinement with SHELXL. Acta Crystallogr. C Struct. Chem..

