# Supplementary material for: A Novel C3/C4-Fused Indole Scaffold through Acid-Catalyzed Cascade Reaction
Source: Molecules. 2024 Jun 27;29(13):3064. doi: 10.3390/molecules29133064 (PMC11243003; doi:10.3390/molecules29133064)

Table S1. Crystallographic Data for Compound **12**

CCDC deposition No.	
Empirical formula	$C_{24}H_{32}N_6O_7$
Formula weight	516.55
Temperature	100(2) K
Crystal system, space group	monoclinic, C2/c
Unit cell dimensions	a = 14.89228(17) Å, α = 90°<br>b = 8.25772(10) Å, β = 95.4293(12)°<br>c = 19.5848(3) Å, γ = 90°
Volume	2397.66(5) $Å^3$
Z; Calculated density	4, 1.431 g/cm^3
Absorption coefficient	0.892 mm^{-1}
F(000)	1096
Crystal size	0.193 x 0.072 x 0.024 mm
Radiation	CuKα (λ = 1.54184 Å)
Theta range for data collection	4.540 to 76.636°
Index ranges	-18<=h<=18, -10<=k<=10, -24<=l<=24
Reflections collected / unique	24294 / 2607 [R(int) = 0.0385]
Data / restraints / parameters	2607 / 0 / 220
Goodness-of-fit on F^2	1.060
Final R indices [I>=2sigma(I)]	R1 = 0.0533, wR2 = 0.1159
R indices (all data)	R1 = 0.0540, wR2 = 0.1152
Largest diff. peak/hole	0.237 and -0.273 e. $Å^{-3}$

Figure S1: Copies of ^{1}H and ^{13}C NMR spectra of Compound **9**.

Methyl 3-formyl-1*H*-indole-4-carboxylate (**9**)

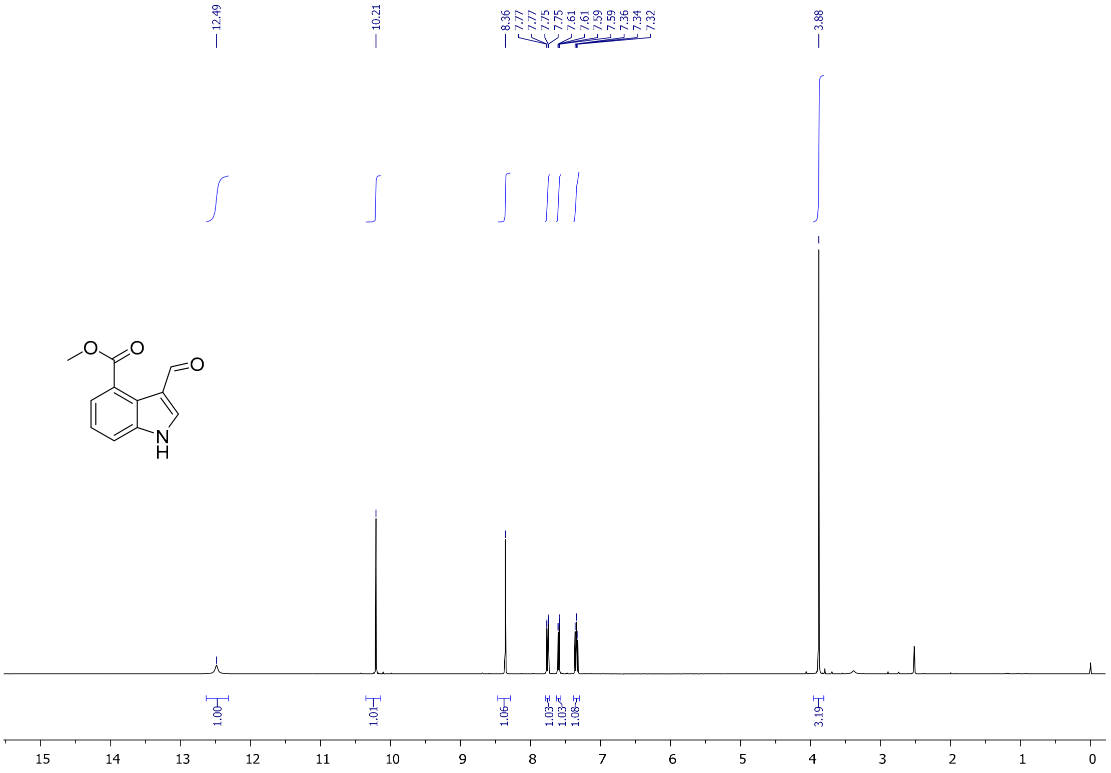

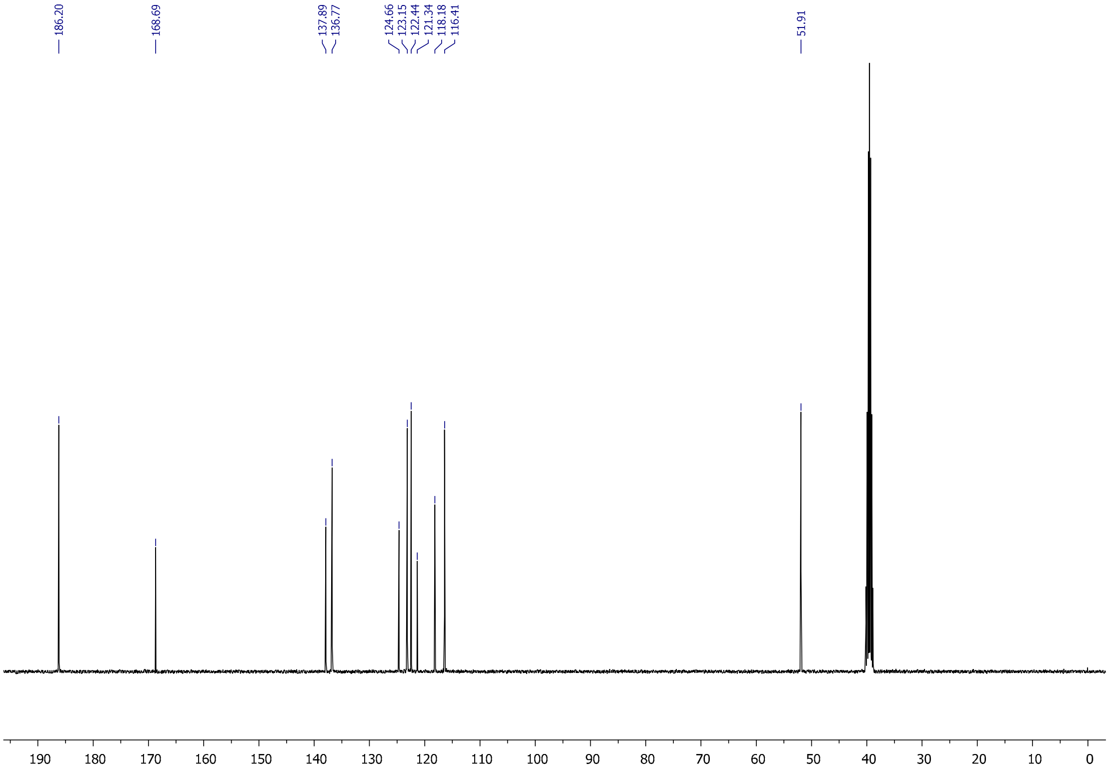

Figure S2: Copies of ^{1}H and ^{13}C NMR spectra of Compound **12**.

8,9,10,10a-Tetrahydroimidazo[1,2-*b*]pyrrolo[4,3,2-*de*]isoquinolin-6(2*H*)-one (**12**)

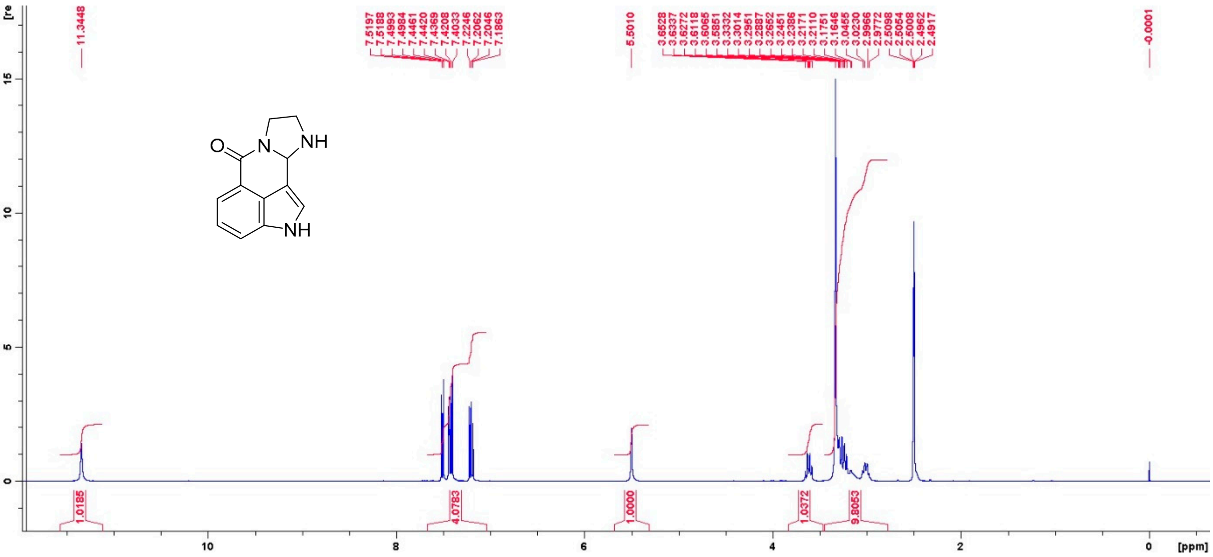

The signal for the four aliphatic protons -CH_2-CH_2- is overlapped by the water signal and thus results in an automatically determined integral labelled 9.8053 (which was not manually corrected to 4.0)

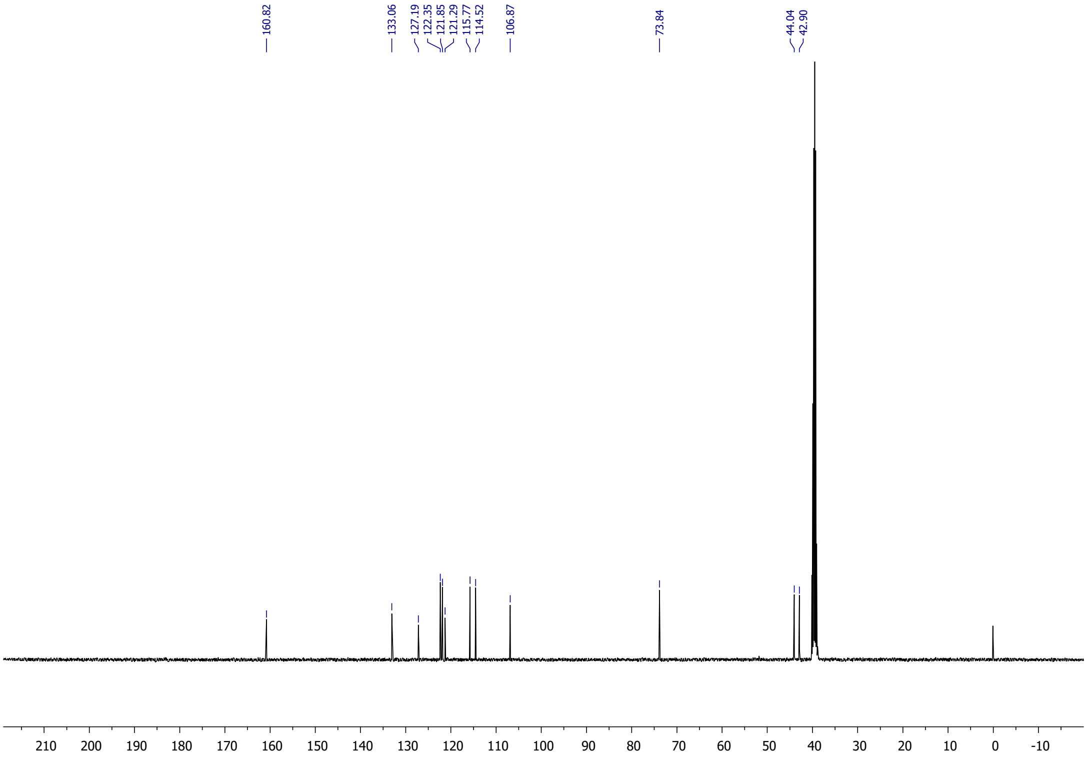

Supplement: Supplementary file 1 [file molecules-29-03064-s001.zip › molecules-3058974-supplementary.pdf]
